# Mechanism–Data Collaboration for Characterizing Sea Clutter Properties and Training Sample Selection

**DOI:** 10.3390/s25082504

**Published:** 2025-04-16

**Authors:** Wenhao Chen, Yong Zou, Zhengzhou Li, Shengrong Zhong, Haolin Gan, Aoran Li

**Affiliations:** School of Microelectronics and Communication Engineering, Chongqing University, Chongqing 400044, China; 20151213039@cqu.edu.cn (W.C.); zouyong@stu.cqu.edu.cn (Y.Z.); 202212021107t@stu.cqu.edu.cn (S.Z.); 202314131173@stu.cqu.edu.cn (H.G.); 202312021018@stu.cqu.edu.cn (A.L.)

**Keywords:** maritime target detection, training sample selection, sea clutter characteristics, model-data-driven, multi-feature fusion

## Abstract

Multi-feature-based maritime radar target detection algorithms often rely on statistical models to accurately characterize sea clutter variations. However, it is a big challenge for these models to accurately characterize sea clutter due to the complexity of the marine environment. Moreover, the distribution of training samples captured from dynamic observation conditions is imbalanced. These multi-features extracted from inaccurate models and imbalanced data lead to overfitting or underfitting and degrade detection performance. To tackle these challenges, this paper proposes a mechanism–data collaborative method using the scattering coefficient as a representative feature. By establishing a mapping relationship between measured data and empirical values, the classical model is piecewise fitted to the measured data. A fusion strategy is then used to compensate for interval discontinuities, enabling accurate characterization of clutter properties in the current maritime environment. Based on the characterized clutter properties, a hybrid feature selection strategy is further proposed to construct a diverse and compact training sample set by integrating global density distribution with local gradient variation. The experiments based on field data are included to evaluate the effectiveness of the proposed method including sea clutter characterization accuracy and training sample selection across various scenarios. Experimental results demonstrate that the proposed method provides a more accurate representation of sea clutter characteristics. Moreover, the detectors trained with the proposed training samples exhibit strong generalization capability across diverse maritime environments under the condition of identical features and classifiers. These achievements highlight the importance of accurate sea clutter modeling and optimal training sample selection in improving target detection performance and ensuring the reliability of radar-based maritime surveillance.

## 1. Introduction

Radar has been widely applied to maritime target detection. However, its performance is constrained in challenging scenarios, such as high sea states, large incident angles, and the detection of weak targets. With the advancements in high-resolution radar and machine learning, traditional energy-based target detection algorithms, such as Constant False Alarm Rate (CFAR), have evolved into multi-feature target detection algorithms. This evolution provides new approaches for addressing complex detection scenarios. Training samples, feature selection, and classification algorithms constitute the three fundamental elements of multi-feature detection, spanning the entire processing cycle [1,2,3]. Common classification features include the normalized Hurst exponent (NHE), relative average amplitude (RAA) [4,5], relative Doppler peak height (RDPH), relative vector entropy (RVE) [6], ridge integral (RI), number of connected regions (NR), and maximum connected region size (MS) [7]. Under the influence of classification methods such as support vector machines (SVMs) [8], convex hull algorithms, and random forests [9], these features contribute to the formation of target detectors.

However, current multi-feature-based target detection methods often overlook maritime conditions and target types, leading to complex nonlinear relationships between the target and clutter. Meanwhile, the large volume of maritime monitoring data is often cluttered and redundant. Studies have shown that an excessive number of redundant training samples significantly increases the complexity of data analysis. Conversely, the absence of key training samples may cause the classifier’s fitted distribution to deviate from the true distribution, thereby reducing detection performance. A significant challenge for multi-feature object detection algorithms is the imbalance in training samples, specifically the mismatch between the data distribution of the training set and the characteristic distribution of sea clutter. This imbalance can lead to both underfitting and overfitting, causing substantial discrepancies in detection performance across different observation scenarios. Therefore, accurately characterizing the properties of sea clutter and selectively acquiring high-quality and representative training samples contribute to the effective discrimination between targets and sea clutter in high-dimensional feature space. This approach can enhance both the detection performance and adaptability of classification algorithms across different scenarios.

To address the challenges of radar-based maritime target detection under adverse sea conditions, a mechanistic and data-driven approach for characterizing sea clutter properties is proposed. The method is based on the theory of metamodeling and selects the backscatter coefficient as the key variable to characterize sea clutter properties. Under the condition of limited measured backscatter coefficient data, an empirical model, combined with the measured data, was used to construct a surrogate equation relating the backscatter coefficient, sea state, and incident angle. This equation was designed to describe the coupling relationship between sea clutter and various environmental factors. Based on this coupling relationship, the method jointly considers both global and local variation trends to select training samples that are comprehensive, cohesive, and compatible, thereby improving the maritime detection performance under different observation conditions.

The remainder of this paper is organized as follows: Section 2 describes the construction process of the sea clutter characterization with the backscatter coefficient as the target variable. Section 3 focuses on the distribution characteristics of the sea clutter, selecting training samples based on its ergodicity and compactness. Section 4 presents experimental analysis of the accuracy of the sea clutter characterization and the effectiveness of the training set, discussing the validity of the proposed method in different scenarios. Section 5 concludes the paper.

## 2. Mechanism–Data Collaboration for Characterizing Sea Clutter Properties

The backscatter coefficient [10] is a key feature of sea clutter, intermittently reflecting the variations and distribution characteristics of sea clutter. Its value is primarily influenced by sea conditions, observation location, and radar polarization characteristics. The interaction between the observational location and sea conditions makes target detection more complex because it exhibits high dimensionality, nonlinearity, and strong coupling, which significantly complicates the mapping relationship between observational conditions and the backscatter coefficient [11].

The surrogate equation is used as an approximate model to describe relatively complex numerical fitting processes, effectively representing the relationship between influencing factors and the objective function. By leveraging meta-models, the traditional model, and measurement data, a surrogate equation can be constructed to describe the highly coupled relationship between the backscatter coefficient and observation conditions, thereby characterizing the properties of sea clutter under different observation conditions.

### 2.1. Characterization of Sea Clutter Properties Under a Single Condition

Representative surrogate equations used in engineering include polynomial response surfaces (PRS), Kriging models, radial basis functions (RBF), artificial neural networks (NNs), support vector regression (SVR), and polynomial chaos expansion (PCE). Polynomial regression surfaces (PRS) is a statistical method that models nonlinear relationships by fitting data to a polynomial function. It is widely used in data fitting and predictive modeling, allowing the estimation of unknown values by capturing complex patterns through higher-degree polynomial terms. The Kriging model [12], based on Gaussian processes (GP), is a flexible probabilistic framework with strong nonlinear fitting capabilities. By using known data, the Kriging model predicts unknown values through interpolation, relying on correlation and model assumptions. The two methods mentioned above have widespread applications in dynamic process modeling, computational materials science, financial time-series fitting, and experimental design.

The PRS model is formulated as

(1) $$y=\beta_{0}+\sum_{i=1}^{k}\beta_{i}x_{i}+\sum_{i<j}\beta_{ij}x_{i}x_{j}+\sum_{i=1}^{k}\beta_{ii}x_{i}^{2}+\epsilon$$

where $\beta =[\beta_{0}\;\beta_{i}\;\beta_{ii}\;\beta_{ij}]$ are parameters to be estimated, x represents the influencing factors that need to be considered, and y denotes the target observation quantity, $\varepsilon \sim N\left(0,\sigma^{2}\right)$.

The Kriging model is formulated as

(2) $$\hat{y}(x)=\beta_{0}+r^{T}(x)R^{-1}(y-\beta_{0}F)$$

where x represents the influencing factors that need to be considered, y denotes the target observation quantity, $\beta_{0}={\left(F^{T}R^{-1}F\right)}^{-1}F^{T}R^{-1}y;\;F=[1\;1\cdots 1{]}^{T}\in R^{n};\;R=\left[\begin{array}{c} R(x^{\left(1\right)},x^{\left(1\right)})\;\cdots \;R(x^{\left(1\right)},x^{\left(n\right)})\\ \vdots \;\;\;\;\;\;\;\;\;\;\;\;\;\;\;\;\;\;\;\;\;\;\;\;\;\;\;\vdots \\ R(x^{\left(n\right)},x^{\left(1\right)})\;\cdots \;R(x^{\left(n\right)},x^{\left(n\right)}) \end{array}\right]\in R^{n*n}$; $r=\left[\begin{array}{c} R(x^{\left(1\right)},x)\;\\ \vdots \\ R(x^{\left(n\right)},x) \end{array}\right]\in R^{n}$. R represents the correlation matrix, and it is composed of the correlation function values between all known samples; r denotes the correlation vector, and it consists of the correlation function values between the unknown point and all known samples.

Sea clutter backscatter coefficients exhibit certain variations, as they are obtained from different collection devices and observational platforms. By utilizing extensive historical data, verification models can partially describe the trend of scatter coefficient variations. Among these, the NRL model [13] is suitable for observational conditions including sea state levels ranging from 1 to 5, incidence angles from 0.1° to 60°, frequencies from 0.5 to 35 GHz, and polarization modes encompassing HH and VV. HH and VV represent co-polarization modes, where HH polarization indicates that the microwave signal oscillates in the horizontal direction, and the receiver also receives the echo signal in a horizontal configuration. VV polarization, on the other hand, signifies that the microwave signal oscillates in the vertical direction, with the receiver configured to receive the echo signal in a vertical orientation. This empirical model is well-suited for high sea states and large incidence angles, and it is expressed as

(3) σdBo=aNRL+bNRL log10⁡(sin⁡∅deg)+(27.5+CNRL∅deg)log10⁡fRF,GHz1+0.95∅deg + dNRL(1+Sss)1(2+0.085∅deg+0.033Sss)+eNRL∅deg2

where $\emptyset_{deg}$ represents the grazing angle, $f_{RF,GHz}$ denotes the carrier frequency, and $S_{ss}$ corresponds to the sea state level; $a_{NRL}$ is a constant; $b_{NRL}$ describes the logarithmic dependence on the incidence angle; $c_{NRL}$ accounts for the effect of radar frequency with additional empirical correction for the incidence angle; $d_{NRL}$ incorporates the impact of sea state with further empirical correction for the incidence angle; and $e_{NRL}$ includes the effect of rapid increase in the scatter coefficient near vertical incidence angles. The parameters are detailed in Table 1.

**Figure 1:**
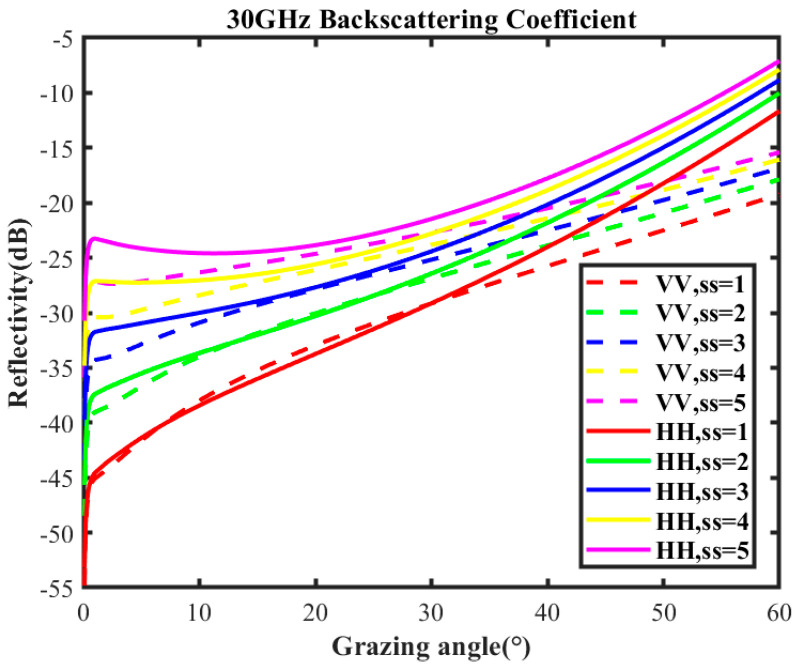
Scattering coefficient of empirical model at 30 GHz carrier frequency.

The scatter coefficient is shown in Figure 1 under the conditions of sea states 1 to 5, HH and VV polarizations, and carrier frequency of 30 GHz.

Due to limitations in data availability, the measured scatter coefficient values in a domestic sea area are obtained with 30 GHz observation radar, and they are shown in Table 2.

**Figure 2:**
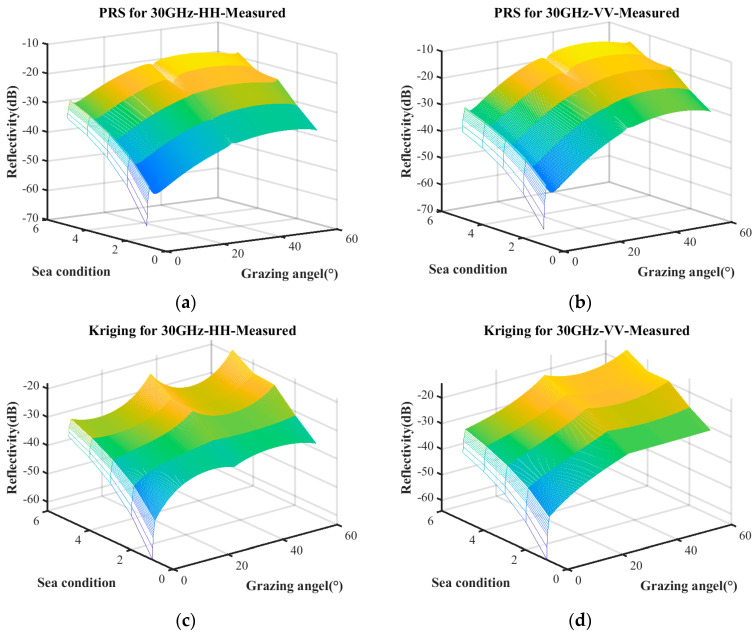
Metamodel constructed from measured data. (**a**) PRS for HH polarization; (**b**) PRS for VV polarization; (**c**) Kriging for HH polarization; (**d**) Kriging for VV polarization.

Based on the distribution of scatter coefficients, polynomial regression and the Kriging model are employed for characteristic fitting. The polynomial regression incorporates quadratic and interaction terms, while the Kriging model utilizes an exponential kernel function. The surface depicted in Figure 2 represents the predicted relationship between reflectivity, sea conditions, and grazing angle. Any point on the surface corresponds to the predicted reflectivity under specific sea conditions and a given grazing angle.

It can be seen in Figure 2 that the sea clutter distributions modeled using PRS and Kriging exhibit a generally consistent trend with that of the empirical model. However, there is significant discontinuity around these measurement data. Therefore, it is necessary to improve the surrogate equation model to meet the accuracy requirement.

### 2.2. Characterization of Sea Clutter Properties Under Mechanism–Data Conditions

The sea clutter scatter coefficient is influenced by multiple factors including observation time, observation location, and measuring instrumentation, and it will inherently have some error compared to traditional empirical models. Meanwhile, its quantity is usually limited. Multiple restrictions make it challenging to construct high-precision surrogate equations for supporting training sample selection.

The principal mapping makes use of the high similarity in overall trends between the empirical models and the measured data, and it allows them to convert to each other under the same influencing factors.

(4) $$\left\{\begin{array}{c} \sigma_{m}^{o}=f(ss,\;\varphi ,\;pola)\\ \hat{\sigma_{r}^{o}}=h(\sigma_{m}^{o}) \end{array}\right.$$

where f represents the surrogate equation constructed based on the NRL model, and h denotes the mapping function.

**Figure 3:**
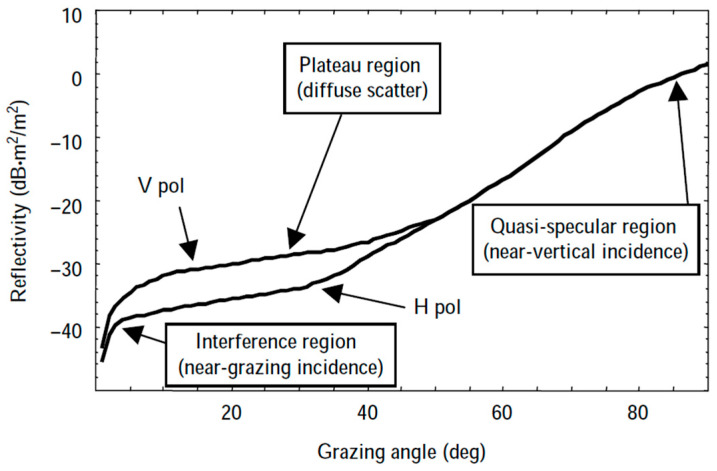
Typical variations of sea clutter scattering coefficient.

The principal mapping assumes that the scatter coefficients estimated by empirical models and the measured data are independently and identically distributed. The correlation mapping between the two scatter coefficients can be directly obtained by the least squares method. However, the sea clutter scatter coefficient varies greatly with different incidence angles, as illustrated in Figure 3. In the quasi-specular region with an incident angle greater than 50°, the backscatter coefficient changes approximately linearly with the incidence angle. In the plateau region with an incidence angle greater than 10° and less than 50°, the backscatter coefficient changes little with the incident angle increasing. In the interference region with the incidence angle of less than 10°, the scatter coefficient decreases sharply.

The relationship between the backscatter coefficient and the grazing angle interval is delineated in Table 3 [14,15].

**Figure 4:**
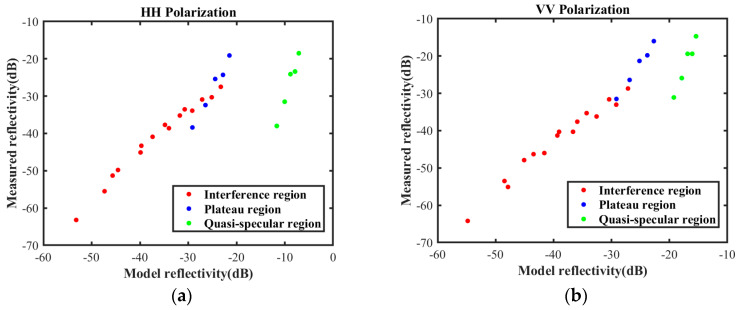
Measured data and empirical model. (**a**) HH polarization; (**b**) VV polarization.

The HH polarization reflectivity and VV polarization reflectivity of measured data and the data predicted by the NRL model are illustrated in Figure 4. All samples are color-coded into three intervals using red, blue, and green, labeled interference region, plateau region, and quasi-specular region, respectively. It is shown that the relationship between the measured data and predicted values across the entire region is not linear, whereas the relationship within each interval is almost linear.

The three segments of the interval are processed using different mapping functions to obtain surrogate equations for the scattering coefficient based on the measured data in each segment. Each segment captures the mechanistic relationship between the scattering coefficient and its influencing factors within the data range. However, discrepancies may exist in the mapping relationship between the measured data and the theoretical data across different segments, leading to discontinuities at points 10° and 30°, which contradict the underlying physical mechanisms. Therefore, interpolation techniques should be employed to address this issue.

By segmenting the incident angle according to different intervals based on the variation range, a piecewise representation of the surrogate equations for the scattering coefficient derived from the measured data is obtained. Additionally, function shifting is applied to align the actual value $\sigma_{r,s_{n}}^{o}$ at the endpoint $\left[0.1{}^{\circ},\;10{}^{\circ}\right],\;\left[10{}^{\circ},\;50{}^{\circ}\right],\;[50{}^{\circ},\;90{}^{\circ}]$ of each mapping interval, ensuring that no discontinuities or step changes occur at the interval endpoints. The resulting shifted surrogate equation for the measured scattering coefficient is as follows:

(5) $$\left\{\begin{array}{c} \hat{\sigma_{r,s_{n}}^{o}}=h_{s_{n}}(\sigma_{m,s_{n}}^{o}),\;n=1,\;2,\;3\\ \hat{\sigma_{r}^{o}}=\hat{\sigma_{r,s_{n}}^{o}}+2\hat{\sigma_{r,s_{1}}^{o}}(10{}^{\circ})+\hat{\sigma_{r,s_{2}}^{o}}(30{}^{\circ})-\hat{\sigma_{r,s_{3}}^{o}}(30{}^{\circ}) \end{array}\right.$$

The measured scatter coefficient and the scatter coefficient estimated by the empirical model principal mapping are denoted as $\left(ss,\;\varphi ,\;pola;\;\sigma_{r}^{o}\right)$ and $\left(ss,\;\varphi ,\;pola;\;\hat{\sigma_{r}^{o}}\right)$ at the same influencing factors, respectively. The difference $∆\sigma_{r,s_{n}}^{o},\;n=1,\;2,\;3$ between $\sigma_{r,s_{n}}^{o}$ and $\hat{\sigma_{r,s_{n}}^{o}}$ at each endpoint of the mapping interval [0.1°, 10°], [10°, 50°], [50°, 90°] can be fitted by the model interpolation function as

(6) $$∆\hat{\sigma^{o}}(x)=b(x{)}^{T}\hat{\beta }+t(x{)}^{T}V^{-1}(y-F\hat{\beta })$$

where $\hat{\beta }=(F^{T}V^{-1}F{)}^{-1}F^{T}V^{-1}y$, $V=[cov(V_{i},V_{j}){]}_{1\leq i,\;j\leq n}=[cov(\sigma_{r}^{o}(x_{i}),\sigma_{m}^{o}(x_{j})){]}_{1\leq i,\;j\leq n}+[\sigma_{\varepsilon }^{2}\eta_{ij}{]}_{1\leq i,\;j\leq n},\;t(x)=[cov(\sigma_{r}^{o}(x_{1}),\;\sigma_{r}^{o}(x)),\cdots cov(\sigma_{r}^{o}(x_{n}),\;\sigma_{r}^{o}(x)){]}^{T}$.

Based on the scattering coefficient surrogate equation derived from the measured data after translation, a combined surrogate equation is formed by incorporating an interpolation function. This approach not only smooths the function to align with the expected theoretical mechanism but also ensures that the equation closely fits the measured data, effectively representing the coupled functional relationship between various influencing factors and the scattering coefficient in the studied sea area.

(7) $$\sigma^{o}=\hat{\sigma_{r}^{o}}+∆\hat{\sigma^{o}}=\hat{\sigma_{r,s_{n}}^{o}}+2\hat{\sigma_{r,s_{1}}^{o}}(10{}^{\circ})-2\hat{\sigma_{r,s_{2}}^{o}}(10{}^{\circ})+\hat{\sigma_{r,s_{2}}^{o}}(30{}^{\circ})-\;\hat{\sigma_{r,s_{3}}^{o}}(30{}^{\circ})+b(x{)}^{T}\hat{\beta }+t(x{)}^{T}V^{-1}(y-F\hat{\beta })$$

Consequently, it effectively characterizes the coupled functional relationships between various influencing factors and the scatter coefficient for the specific sea area.

## 3. Selection of Training Samples Based on the Characterization of Sea Clutter Properties

The training samples selected for maritime radar target detection based on the surrogate equation should meet three requirements: completeness, density, and compatibility. The completeness mainly considers the value range of the influencing factors, which should be covered as comprehensively as possible. The density focuses on the number of training samples, which could capture the classification trends as refined as possible. The compatibility is not taken into account for maritime target detection and is considered as an anomaly relative to sea clutter.

### 3.1. Method to Satisfy Ergodicity Criterion

The non-uniform and non-smooth regions in the surrogate equation can characterize the trend of sea clutter characteristics, and it is the key areas that are the focus of the training sample selection. The global density algorithm based on Voronoi partitioning [16] can determine the sampling density in the vicinity of training samples by assessing the size of the Voronoi cells, but it sometimes ignores the region’s fluctuations. In contrast, the local nonlinearity algorithm based on gradient estimation can identify the regions with higher nonlinearity and focus, but it has weaker global exploration capabilities. This paper proposes a combination of the global density algorithm and the local nonlinearity algorithm to select training samples satisfying the criterion of completeness.

#### 3.1.1. Global Density Algorithm Based on Voronoi Partitioning

For the global exploration criterion, a Voronoi-based algorithm partitions the surface model and estimates sample distribution density. On the surrogate equation surface, random samples—typically 100 times the measured data—are generated around observations. Distances between random and measured points are computed sequentially, ranking measured data by their nearest random samples. The relative proportion of random samples approximates the polygonal area. The algorithm’s pseudocode is provided below (Algorithm 1).


**Algorithm 1** Estimation of the Area of Voronoi Polygons1S←|X|×100 random samples in the domain2

V←[0, 0, …, 0]

3

for all s∈S do

4 
dis←∞
5   
for all x∈X
6        
if∥x−s∥ < d then
7             
$x_{closet}\leftarrow x$
8             
dis←∥x−s∥
9             
end if
10      
end for
11      
$V\left[x_{closet}\right]\leftarrow V\left[x_{closet}\right]+(\frac{1}{\left|s\right|})$
12

end for




Where dis represents the distance between the two samples, X denotes the measured samples, V is the vector of polygonal areas corresponding to X, and $\left|P\right|$ indicates the number of measured data samples. S refers to the selected set of random samples, $\left|S\right|$ is the number of selected random samples, S symbolizes an individual random point, and $x_{closet}$ denotes the measured data point closest to the current random point.

#### 3.1.2. Local Nonlinearity Algorithm Based on Gradient Estimation

To ensure comprehensive coverage, a large sample set is required due to the surrogate equation surface’s uneven distribution, regional nonlinearity variations, and challenges in fitting high-linearity areas. After Voronoi-based global partitioning, regional nonlinearity is estimated via gradient changes. Voronoi divides the surface into multiple regions containing numerous samples, from which candidate training samples $N(x_{m})=\left\{x_{m1},x_{m2},\cdots x_{mn}\right\}$ are selected based on clustering and exclusion strength. Clustering strength, defined as the average distance between candidates and their region’s center, indicates how well samples concentrate near the center, enhancing information density.

(8) $$C(N(x_{m}))=\frac{1}{n}\sum_{i=1}^{n}\left\|x_{mi}\right\|$$

Exclusion strength, defined as the average minimum distance between regional samples, evaluates their uniform distribution around the center. Greater uniformity enhances gradient variation information.

(9) $$R(N(x_{m}))=\frac{1}{n}\sum_{i=1,\;i\neq j}^{n}min\left\|x_{mi}-x_{mj}\right\|$$

where $x_{m}$ represents the segmented region, n denotes the number of candidate samples within the region, and $x_{mj}$ refers to the j-th candidate point of $x_{m}$.

When candidate samples share equal clustering strength, they are randomly distributed on a circle centered at the region’s center. Selecting *n* samples that maximize exclusion strength determines the regional candidate set. This set should closely approximate the intersecting polyhedron, with a shorter distance to the center being preferable. The similarity measurement equation is defined as

(10) $$K(N(x_{m}))=\frac{S(N(x_{m}))}{C(N(x_{m}))},\;d>1$$

where d denotes the spatial dimension. According to the measurement formula, if a regional candidate sample point is $S(N(p_{r}))=1$, it represents a perfect intersecting polyhedron region.

After selecting the regional candidate samples, a hyperplane is constructed to fit the candidate samples by means of the least squares method. The nonlinearity of the region ${\;x}_{m}$ can be calculated and normalized as follows:

(11) $$E(x_{m})=\sum_{i=1}^{n}\left|f(x_{mi})-(f(x_{m0})+g_{m}(x_{mi}-x_{m0}))\right|$$

(12) $$NE(x_{m})=\frac{E(x_{m})}{\sum_{i=1}^{n}E(x_{i})}$$

where $x_{m0}$ represents the central point of region $x_{m}$, and $NE(x_{m})$ denotes the normalized nonlinearity.

#### 3.1.3. Training Sample Point Value Selection Under Dual Constraints of Global Density and Local Nonlinearity

After computing the size of the Voronoi cells, these samples are selected according to the criteria of larger Voronoi cell size and lower density, where a large number of candidate training samples are then randomly selected. The density and nonlinearity of all candidate samples are synthesized and sorted in descending order to obtain the newly added training sample point values and build the candidate training samples.

(13) $$H(x_{m})=V(x_{m})+NE(x_{m})$$

where $V(x_{m})$ and $NE(x_{m})$ represent regional density and nonlinearity, respectively. Both metrics were standardized within their respective ranges.

### 3.2. Method to Satisfy Compactness Criterion

The compactness is an index for assessing the adequacy of the training samples, and it determines whether a theoretically selected training sample can effectively cover the entire surrogate equation surface. The effectiveness is that the entire surrogate equation surface can be filled with coverage circles, where each training sample point is set as the center with an appropriate radius.

The compactness of the training samples is evaluated by constructing the minimum coverage radius as the metric Φ. Considering the characteristics and the ranges of influencing factors such as incident angle and sea conditions, the distance between any two samples is defined as

(14) $$d(x_{1},x_{2})=\{(x_{1,\varphi }-x_{2,\varphi }{)}^{2}+\frac{1}{5^{2}}[1-\delta_{1,ss}(x_{2,ss})]{\}}^{\frac{1}{2}}$$

where *x*_1,φ_ is the incident complementary angle whose value range is normalized to [0, 1] using the standard Euclidean distance. $\delta_{i,ss}(x_{i,ss})$ is the Dirac delta function, which is 1 when $x_{1,ss}=x_{2,ss}$ is true and 0 otherwise. The sea conditions were assessed on a scale of 1 to 5.

The minimum covering radius is defined as

(15) $$r(X_{0})=\frac{1}{2}\underset{x_{1}\in X}{\max}\;\underset{x_{2}\in X_{0}}{\min}d(x_{1},x_{2})$$

where $X_{0}$ denotes the theoretically selected training samples and X represents the set of all possible samples for the surrogate equation.

The coverage area is denoted as

(16) $$\Phi (X_{0})=S({⋃}_{i=1}^{n}B(x_{0}^{i},\;r(X_{0}))),\;x_{0}^{i}\in \;X_{0}$$

where $\Phi (X_{0})$ indicates the coverage area corresponding to the theoretical training samples $X_{0}$, while $B(x_{0}^{i},\;r(X_{0}))$ represents the circular area in the theoretical training samples $X_{0}$ with center $x_{0}^{i}$ and radius $\;r(X_{0})$.

The samples with high density could be selected by the following method (Algorithm 2).
**Algorithm 2** Selection of an ideal training sample that satisfies density requirementsInput:Original measured data set X, number of samples in the original measured data set N, candidate training samples $\xi_{n}$, number of samples in the candidate training samples J, surface area of the surrogate equation S, and coverage ε
1:Count $\Phi \left(X\right)$, j←1
2:WHILE (j≤J) do3:From the difference set $\xi_{n}\setminus X$ between the original measured data set and the candidate training samples, sort according to the dual constraints of global density and local nonlinearity. This process yields the top 5 samples, denoted as {$x_{1}\}$.4:Add ${\{x}_{1}\}$ to the original measured data set X to form the theoretical training samples $X\prime =\left\{x_{1}\right\}\cup X$, and simultaneously update X, make X= X′
5:Count $\Phi \left(X\prime \right)$
6:If $\Phi \left(X\prime \right)/S\geq \varepsilon$, break7:ENDWHILEOutput:If j<J, output the theoretical training samples X; If j=J, $\xi_{n}$ is insufficient.

### 3.3. Transformation from Training Samples into Measurements

After obtaining training samples with the theoretical values satisfying ergodicity and sample sizes meeting compactness, it is necessary to optimize and adjust the ideal training samples based on the detector’s acquisition capabilities and the influencing factor partition units, and then transform the training samples into the actual measurements.

For influencing factors of the actual measurements, the quantified units of incidence angle and sea condition are 1° and level 1, respectively. An ideal sample point would be adjusted to the practical training sample point under the condition that their influencing factors are within the same interval range. When the angular resolution of radar data acquisition is excessively large (theoretically exceeding 3°), the theoretical training samples should be mapped to their corresponding angular intervals. In this case, it is advisable to construct the training dataset by randomly selecting valid samples from the collected data in proportion to the number of theoretical samples assigned to each interval, thereby ensuring consistency with the distribution under the specified acquisition conditions.

After revising the training sample point, each training sample point requires multiple valid sets of sea clutter training data. If the sea clutter data are insufficient or unavailable, a field experiment under corresponding conditions should be conducted to collect more measured data, or the sea clutter training data closest to the ideal sample should be selected to complete the samples.

## 4. Results and Discussion

To validate the effectiveness of the proposed method, it is necessary to make use of the selected training samples to test both the constructed surrogate equation and the radar maritime target detection. The surrogate equation is a key prerequisite for training sample selection, and the training samples are a critical factor influencing the target detection performance. The measurement area in this study is Langya Taiwan, located in Qingdao, Shandong Province, China. The backscatter coefficient of sea clutter is measured and provided by specialized research institutes focused on target characteristics. The data acquisition equipment consists of two independent maritime surveillance radars. Both radars operate in a coherent system and transmit linear frequency-modulated (LFM) pulse signals in Ka-band with a carrier frequency of 30 GHz. The only difference between them is their polarization: one utilizes HH polarization, and the other adopts VV polarization. In search mode, the operating bandwidth is 10 MHz, the range resolution is 15 m, and the maximum detection range is 50 km. The search angle covers ±45°, and the radars are capable of staring operation at predefined angles.

### 4.1. Accuracy Analysis of Sea Clutter Characterization

Based on measured data and the empirical model, sea clutter characteristics are constructed under three scenarios: using PRS, using Kriging, and combining measured data with the empirical model. To evaluate the accuracy of the proposed characterization, a cross-validation method is employed, allowing for the selection of the model with superior predictive performance to determine the optimal training samples.

**Figure 5:**
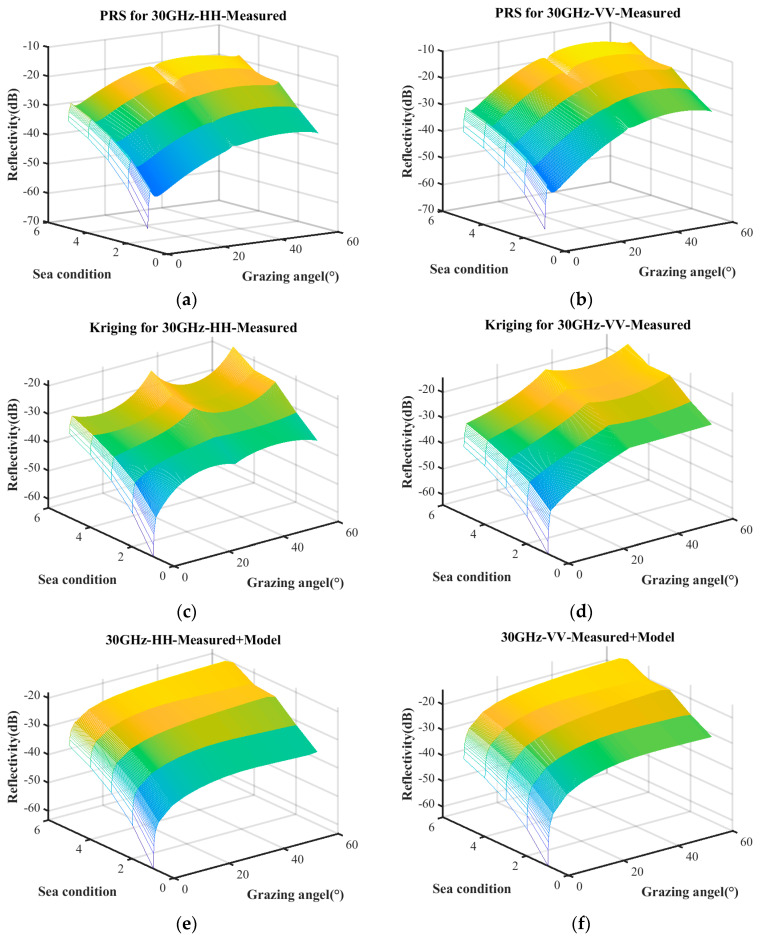
Comparison of sea clutter characterizations constructed by three methods. (**a**) HH polarization based on measured data by PRS; (**b**) VV polarization based on measured data by PRS; (**c**) HH polarization based on measured data by Kriging; (**d**) VV polarization based on measured data by Kriging; (**e**) HH polarization based on empirical model and measured data; (**f**) VV polarization based on empirical model and measured data.

Figure 5 presents the sea clutter characterizations constructed under these three conditions. Figure 5a–d represent the predictive results obtained solely from measured data using the PRS and Kriging model, whereas Figure 5e,f refer to the predictions derived from the combination of the empirical model and measured data. The surface plot illustrates the predictive relationship between reflectivity, grazing angle, and sea conditions. Notably, the characterization based on measured data exhibits significant differences compared to the one incorporating both the empirical model and measured data.

**Figure 6:**
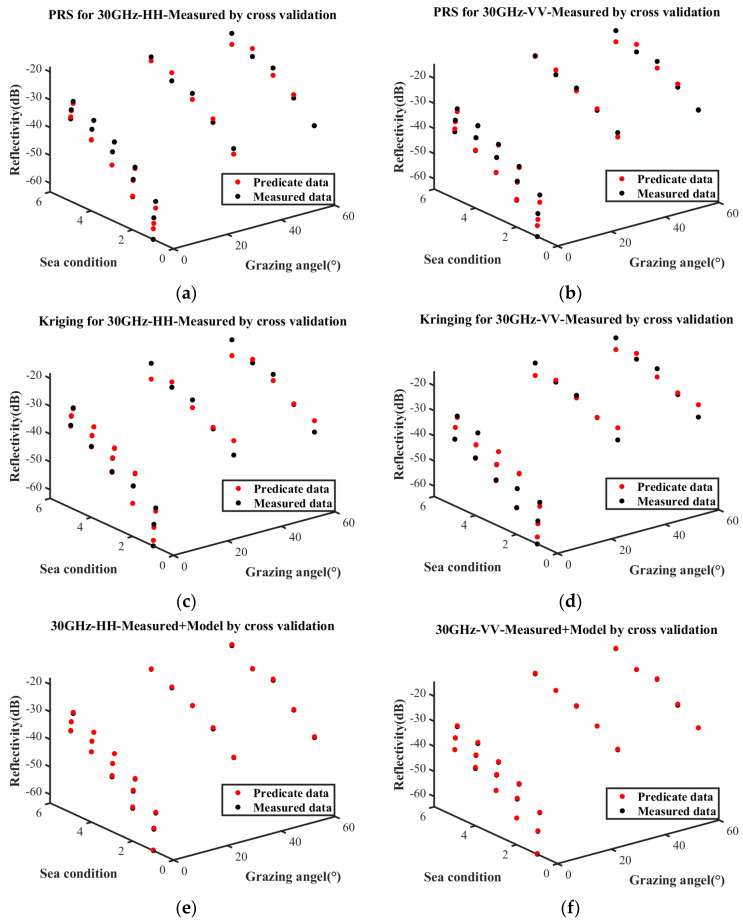
Difference distributions of surrogate equations. (**a**) PRS for HH polarization based on measured data; (**b**) PRS for VV polarization based on measured data; (**c**) Kriging for HH polarization based on measured data; (**d**) Kriging for VV polarization based on measured data; (**e**) HH polarization based on empirical model and measured data; (**f**) VV polarization based on empirical model and measured data.

Cross-validation was employed to evaluate the three surrogate equations, and the validation results are presented in Figure 6. Each point represents the reflectivity under various sea conditions and grazing angles, wherein the red points and black points indicate the predicted values and actual values, respectively. The closer the red points and black points are under the same sea conditions and incidence angles, the more accurate the prediction results.

The metrics used to evaluate the predictive performance are the mean squared error (MSE), root mean squared error (RMSE), mean absolute error (MAE), and $R^{2}$. The evaluation metrics of the sea clutter characterization based on the measured data and the combination of the measured data with the empirical model are presented in Table 4. When relying solely on measured data, the PRS demonstrates better performance than the Kriging model in fitting the distribution of sea clutter characteristics. Compared to the sea clutter characterization based on measured data, the sea clutter characterization based on a combination of the empirical model and measured data has smaller MSE, RMSE, and MAE, and its $R^{2}$ is closer to the ideal value of 1. These indices indicate that the equation based on the measured data has poorer fitting accuracy.

By comparing these accuracy metrics, it is evident that the sea clutter characterization based on the combination of measured data and empirical model significantly outperforms that based on only measured data. The former can more accurately describe the characteristics of the observed sea surface, and its training samples would be more suitable for the marine region.

### 4.2. Target Detection on Different Coverage Training Samples

The position and the quantity of samples are the primary factors in training sample selection. The position is selected based on the fluctuation intensity of the sea clutter, and the quantity of samples is determined by the chosen coverage rate. The number of samples directly determines the volume and the effectiveness of the training samples.

**Figure 7:**
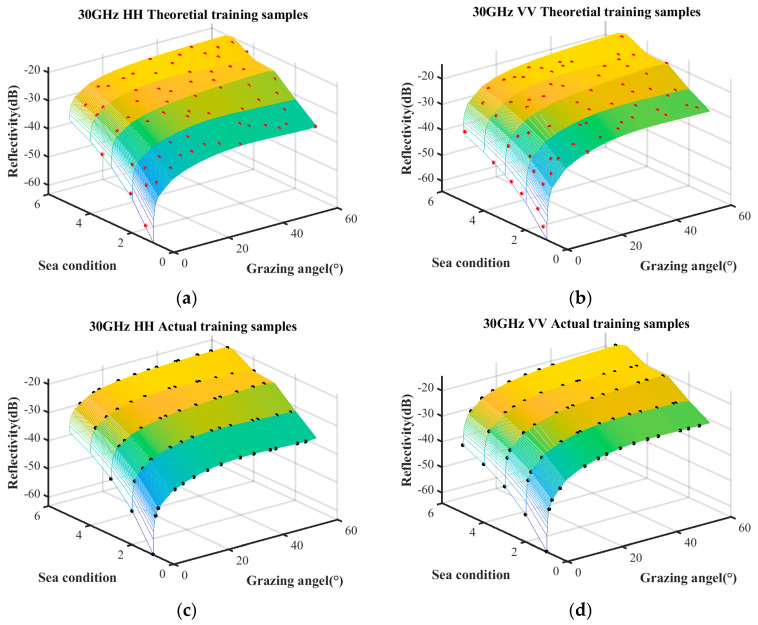
Training samples with coverage rate of 85%. (**a**) HH polarization in theoretical training samples; (**b**) VV polarization in theoretical training samples; (**c**) HH polarization in actual training samples; (**d**) HH polarization in actual training samples.

**Figure 8:**
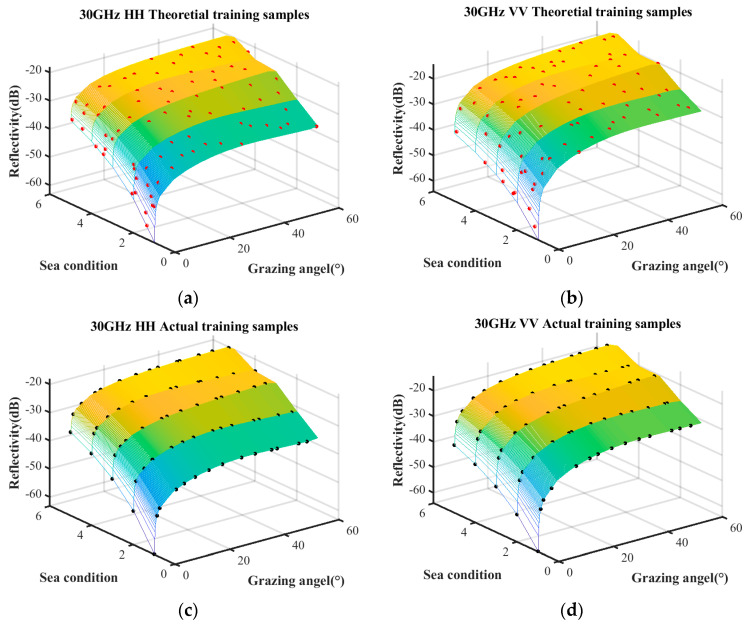
Training samples with coverage rate of 90%. (**a**) HH polarization in theoretical training samples; (**b**) VV polarization in theoretical training samples; (**c**) HH polarization in actual training samples; (**d**) VV polarization in actual training samples.

**Figure 9:**
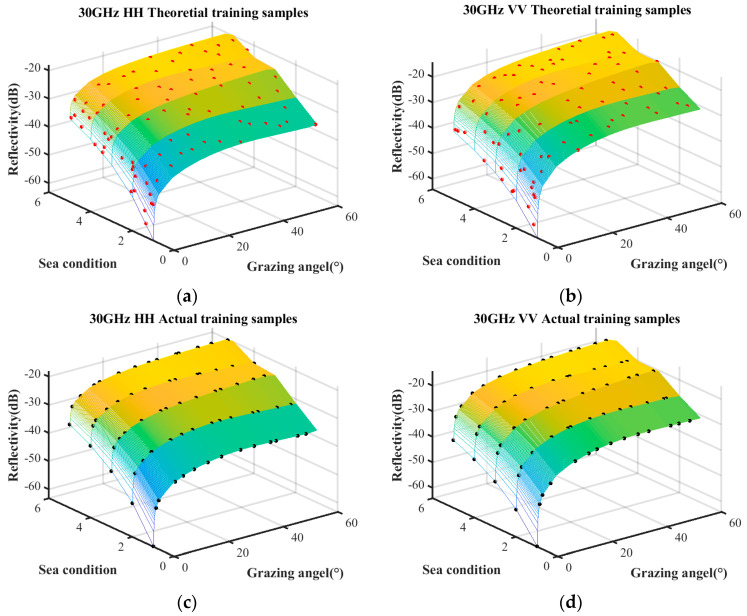
Training samples with coverage rate of 95%. (**a**) HH polarization in theoretical training samples; (**b**) VV polarization in theoretical training samples; (**c**) HH polarization in actual training samples; (**d**) VV polarization in actual training samples.

The sample quantities under different coverage rates are discussed first. The trained samples under the condition of a coverage rate of 85%, 90%, and 95% are selected by the surrogate equation based on the combination of measured data and empirical model. The theoretical training samples and the actual training samples are shown in Figure 7, Figure 8 and Figure 9, respectively. The surface represents the predictive relationship between reflectivity, sea condition, and grazing angle, wherein the red points and black points denote theoretical sample points and actual sample points, respectively.

The sample quantities of the theoretical training samples and the actual training samples under the aforementioned three coverage conditions are listed in Table 5.

With the increase in coverage rate, the theoretical sample quantity increases linearly, while the actual sample approaches saturation when the coverage rate reaches 90%. Additionally, compared to the coverage increase from 90% to 95%, the coverage increase from 85% to 90% obtains a significantly greater number of theoretical samples. Therefore, the coverage rate of 90% minimizes substantial wastage of theoretical samples while ensuring that the actual samples are sufficiently abundant.

After obtaining the training samples, the common time-frequency features are selected for a multi-feature detector to detect maritime targets. These features include common ridge integral (RI), number of connected regions (NR), and maximum connected region size (MS). For each of the three coverage rate training samples, these time-frequency features are combined with a single-class SVM classifier (OneClass-SVM) [17] to obtain the corresponding feature classifiers. The radial basis function (RBF) is used as the kernel, with the hyperparameter ν set to 0.05, the RBF kernel parameter γ set to $\frac{1}{var(RI)+var(NR)+var(MS)}$, and other parameters, such as tol (convergence tolerance), set to their default values. To cover a wide range of maritime scenarios as comprehensively as possible, five types of scenarios are selected based on the available measured data, and they are listed in Table 6: conventional ships under routine sea conditions, small fishing boats and sea surface floats under routine sea conditions, cruise ships under high sea conditions, conventional ships under large incidence angles, and cruise ships under both large incidence angles and high sea conditions.

For each scenario condition, 500 coherent processing interval (CPI) pulse compression data points in the Ka-band are selected to test the classifier performance. Every CPI includes 64 pulse echoes. After performing coherent accumulation on each CPI, a detection threshold set at 9 dB is used to identify suspicious target regions. The corresponding echo information from these regions is then extracted and input into a feature classifier to determine the attributes of the suspicious areas. The detection probability is defined as the ratio of correctly detected targets to the total number of actual targets, while the false alarm probability is defined as the ratio of falsely identified targets to the total number of suspicious locations. The test results are presented in Table 7. Classifier 1, Classifier 2, and Classifier 3 represent the trainer selected under 85% coverage, 90% coverage, and 95% coverage, respectively.

It can be seen from Table 7 that the performance of the classifier for conventional ship detection under routine sea condition with the coverage rate of 85% is similar to that with the coverage rate of 90%. The performance of conventional ship detection scenarios under large incidence angles also remains nearly unchanged. However, the classifier with the coverage rate of 85% shows slightly weaker performance in detecting small targets under routine sea conditions, cruise ships under high sea conditions, and cruise ships under both large incidence angles and high sea conditions. Meanwhile, the detection performance with a coverage rate of 90% is equivalent to that with a coverage rate of 95% in all aspects. From the perspective of detection performance, classifiers operating at a coverage rate of 90% are better suited for various application scenarios.

In summary, the performance of the detector with a coverage rate of 85% is somewhat inferior to that obtained with a coverage rate of 90% or higher, particularly in more complex observational scenarios. To enhance the environmental adaptability of the detector while minimizing the excessive waste of theoretical samples, it is recommended to choose a coverage rate of 90% for training samples.

### 4.3. Target Detection on Different Training Sample Selection Methods

The detection performance is evaluated by comparing feature classifiers constructed using different training sample selection methods, including partition strategies [18], expert knowledge [19], K-means clustering [20], and deep learning methods such as self-organizing maps (SOMs) [21]. The training sample selection methods and classifier training time are listed in Table 8. The training datasets for the seven classifiers are constructed based on different sampling strategies. Excluding the time required for experimental data acquisition, the classifier training time is defined as the average duration of 10 repetitions of training the classifier using a large set of routinely collected radar data as input, with MATLAB 2021(a) (requiring the DACE toolbox 2.0 and LibSVM toolbox 2.88) and PyTorch 1.8.0.

Classifier A: The training dataset is generated using the proposed method, selecting 70 training sample positions based on a 90% coverage criterion. From each position, 10 mid-frequency echoes are chosen, resulting in a total of 700 mid-frequency echoes.

Classifier B: This dataset expands upon that of Classifier A by arbitrarily adding 30 additional training sample positions along with their corresponding mid-frequency echoes, introducing redundancy relative to Classifier A.

Classifier C: The training dataset is formed by randomly selecting 70% of the data from a large collection of routinely acquired observational echoes, leading to an exceptionally large sample size.

Classifier D: The dataset consists of 700 mid-frequency echoes, obtained by randomly selecting 140 samples from each of the five predefined test scenarios.

Classifier E: The training dataset is constructed using K-means clustering, where the number of clusters is determined based on the within-cluster sum of squares (SSE). A total of 70 sample positions are selected using the Euclidean distance metric.

Classifier F: The dataset is created by uniformly sampling 50 points from the surrogate equation, which serve as input to the self-organizing map (SOM). Based on this, 70 sample locations are selected to form the training dataset.

Classifier G: This dataset is derived from measured reflection coefficients, where 70 sample positions are identified using SOM to construct the final training set.

Classifier D requires approximately 4 s for model construction, with the time spent on training sample selection being negligible. The primary computational cost lies in feature extraction and classifier training. Classifiers A and B represent the proposed methods. Classifier A builds upon the process of Classifier D by incorporating surrogate equation construction and targeted training sample selection, which adds around 5 s to the overall time. Classifier B further extends Classifier A by introducing a small number of additional training samples.

Classifiers F and G utilize deep learning-based strategies for training sample acquisition. Classifier F performs training based on samples drawn from the surrogate equation of Classifier A, resulting in approximately five additional seconds of training time. Classifier G, in contrast, uses 25 measured data points as input and therefore incurs a relatively lower computational cost.

Classifiers C and E involve clustering procedures that are dependent on the total number of samples. For example, applying K-Means to 1000 frames takes about 2 s, while training a classifier on 700 selected frames requires approximately 7 s.

In summary, the training time for all methods does not exceed 12 s, demonstrating favorable feasibility for practical engineering applications.

**Figure 10:**
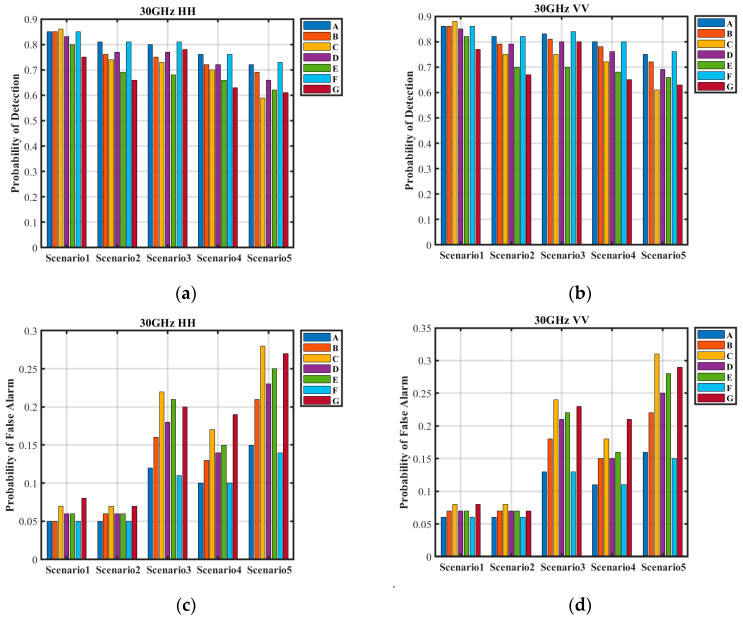
Detection performance of four classifiers. (**a**) Detection probabilities under HH polarization. (**b**) Detection probabilities under VV polarization. (**c**) False alarm probabilities under HH polarization. (**d**) False alarm probabilities under VV polarization.

The detection performance of the seven classifiers across five different scenarios is presented in Figure 10. Among these scenarios, conventional ships operating under routine sea conditions are classified as simple scenarios. In contrast, small targets under routine sea conditions, cruise ships under high sea states, and conventional ships at large incidence angles are categorized as complex scenarios. Finally, cruise ships subject to both large incidence angles and high sea states are considered difficult scenarios.

Table 9 presents the detection probabilities and false alarm rates of the seven classifiers across five scenarios. The proposed method achieves consistently high detection probabilities and low false alarm rates in all cases. In the simple scenario, the detection probability exceeds 0.85, while the false alarm rate remains below 0.06. In the complex scenario, except for the HH polarization condition under large grazing angles (detection probability of 0.76), all detection probabilities exceed 0.8. The false alarm rates are approximately 0.6 for weak targets, 0.13 under high sea state conditions, and 0.11 at large grazing angles. In the difficult scenario, the detection probability is around 0.72, with a false alarm rate of approximately 0.15.

Compared to the proposed method, Classifier B exhibits lower detection probabilities in all scenarios except the simple one, with increased false alarm rates across the board. This decline is attributed to the inclusion of redundant samples, which distort the training sample distribution and reduce the classifier’s ability to accurately distinguish sea clutter characteristics.

Classifier C achieves the highest detection probability in the simple scenario, reaching 0.86 (HH) and 0.88 (VV). However, in the other four scenarios, its detection probabilities are lower than those of the proposed method, and it exhibits the highest false alarm rates across all scenarios. Moreover, its overall detection performance deteriorates further compared to Classifier B. The excessive inclusion of conventional samples may cause the classifier to overlook critical features in complex and difficult scenarios. These results underscore that training sample quality is more crucial than sheer quantity, emphasizing the importance of maintaining a balanced sample distribution.

Both Classifier D and Classifier E demonstrate lower detection probabilities and higher false alarm rates than the proposed method across all scenarios, leading to a notable performance decline. The reliance on expert judgment in sample selection proves less effective than the proposed surrogate equation, particularly in scenarios where negative samples are essential for anomaly detection, as capturing their variations is critical. Similarly, while K-means clustering is generally effective for sample selection, its dependence on the existing data distribution makes it vulnerable to sample imbalance, potentially resulting in skewed training sets.

Classifiers F and G integrate deep learning for sample selection. Classifier F performs worse than Classifier C in the simple scenario but achieves the highest detection probabilities in the remaining four scenarios. By employing large-scale sampling from a surrogate equation as input and using self-organizing maps (SOMs) for sample selection, Classifier F effectively captures sea clutter variations. This results in superior performance compared to traditional methods that separately consider global and local changes. However, due to limitations in acquisition equipment and environmental factors, its overall performance remains comparable to that of Classifier A. In contrast, Classifier G exhibits the lowest detection performance among the seven classifiers. In the difficult scenario, its detection probability drops to 0.61 (HH) and 0.63 (VV), while its false alarm rate increases to 0.27 (HH) and 0.29 (VV). The limited number of input samples for SOM leads to a substantial misrepresentation of sea clutter variations, causing the training samples to deviate significantly from the actual distribution and severely impairing detection performance.

The proposed method selects training samples by integrating observational data with empirical models, without relying on the target data distribution. It demonstrates robust detection performance across diverse scenarios, effectively adapting to the complex and dynamic maritime environment. Furthermore, the method maintains both the interpretability of sample selection and the effectiveness of training samples, ensuring reliable performance in practical applications.

## 5. Conclusions

This study presents a target detection training sample selection method based on a model-data-driven approach to detect maritime radar targets. The experimental results on Ka-band radar sea clutter measured data demonstrate that the proposed method could select effective training samples by means of the combination of measured data with the empirical model. The proposed method achieves better performance in radar target detection under different sea conditions, and it significantly enhances maritime observation capabilities.

Moreover, the proposed method can improve detection performance without significant modifications to existing software algorithms and hardware conditions. Additionally, this method is not restricted to a given maritime region and can be broadly applied to any sea condition.

## Figures and Tables

**Table 1:** Parameters of NRL model.

Polarization	$a_{NRL}$	$b_{NRL}$	$c_{NRL}$	$d_{NRL}$	$e_{NRL}$
HH	−73	20.78	7.351	25.65	0.0054
VV	−50.79	25.93	0.7093	21.58	0.0021

**Table 2:** (**a**). Measured data under HH polarization mode. (**b**). Measured data under VV polarization mode.

(**a**)					
**Grazing Angle** **Sea Conditions**	**0.1°**	**0.3°**	**1.0°**	**30°**	**60°**
1	−63.2	−55.5	−49.8	−38.4	−38.0
2	−51.3	−45.1	−40.9	−32.4	−31.5
3	−43.3	−38.6	−35.2	−25.4	−24.1
4	−37.7	−33.9	−30.9	−24.3	−23.4
5	−33.54	−30.3	−27.5	−19.1	−18.5
(**b**)					
**Grazing angle** **Sea conditions**	**0.1°**	**0.3°**	**1.0°**	**30°**	**60°**
1	−64.2	−55.1	−47.9	−31.5	−31.1
2	−53.5	−46.0	−40.3	−26.4	−25.9
3	−46.3	−40.3	−35.3	−21.3	−19.4
4	−41.3	−36.2	−31.6	−19.8	−19.4
5	−37.6	−33.03	−28.7	−16.0	−14.7

**Table 3:** The correspondence between region and grazing angle interval.

Region	Grazing Angle Interval
S1 Interference region	φ ≤10°
S2 Plateau region	10° < φ ≤ 50°
S3 Quasi-specular region	50°<φ

**Table 4:** Accuracy metrics of surrogate equations.

		MSE	RMSE	MAE	*R* ^2^
HH	PRS	3.1662	1.7794	1.2807	0.9739
	Kriging	5.3245	2.3075	1.4321	0.9562
	Combination	2.3440	1.5310	0.7626	0.9946
VV	PRS	3.3024	1.8172	1.2764	0.9795
	Kriging	4.9584	2.2267	1.3927	0.9692
	Combination	3.1847	1.7846	0.8820	0.9951

**Table 5:** Sample quantities under different coverage rates.

Polarization	Coverage Rates	Sample Quantity of Theoretical Training Samples	Sample Quantity of Actual Training Samples
HH	85%	70	65
	90%	85	70
	95%	90	70
VV	85%	65	62
	90%	75	70
	95%	80	70

**Table 6:** Correspondence table of test scenario numbers and descriptions.

Test Scenario Number	Test Scenario Description
Scenario 1	Conventional ships under routine sea conditions
Scenario 2	Small fishing boats, sea surface floats, and other small targets under routine sea conditions
Scenario 3	Cruise ships under high sea conditions
Scenario 4	Conventional ships under large incidence angles
Scenario 5	Cruise ships under both large incidence angles and high sea conditions

**Table 7:** (**a**). Detection probabilities of three classifiers under HH polarization. (**b**). Detection probabilities of three classifiers under VV polarization. (**c**). False alarm probabilities of three classifiers under HH polarization. (**d**). False alarm probabilities of three classifiers under VV polarization.

(**a**)					
**Classifier**	**Scenario 1**	**Scenario 2**	**Scenario 3**	**Scenario 4**	**Scenario 5**
Classifier 1	0.85	0.80	0.79	0.76	0.70
Classifier 2	0.85	0.81	0.80	0.76	0.72
Classifier 3	0.85	0.81	0.80	0.76	0.72
(**b**)					
**Classifier**	**Scenario 1**	**Scenario 2**	**Scenario 3**	**Scenario 4**	**Scenario 5**
Classifier 1	0.86	0.82	0.81	0.81	0.73
Classifier 2	0.86	0.82	0.83	0.81	0.75
Classifier 3	0.86	0.82	0.83	0.81	0.75
(**c**)					
**Classifier**	**Scenario 1**	**Scenario 2**	**Scenario 3**	**Scenario 4**	**Scenario 5**
Classifier 1	0.06	0.06	0.13	0.11	0.17
Classifier 2	0.05	0.05	0.12	0.10	0.15
Classifier 3	0.05	0.05	0.12	0.10	0.15
(**d**)					
**Classifier**	**Scenario 1**	**Scenario 2**	**Scenario 3**	**Scenario 4**	**Scenario 5**
Classifier 1	0.08	0.08	0.15	0.13	0.18
Classifier 2	0.06	0.06	0.13	0.11	0.16
Classifier 3	0.06	0.06	0.13	0.11	0.16

**Table 8:** Classifiers based on training sample selection methods.

Classifier	Training Sample Selection Methods	Classifier Training Time (in Seconds)
Classifier A	Method proposed in this paper	About 9 s
Classifier B	Method proposed in this paper, adds redundant samples	About 10 s
Classifier C	Method based on partitioning strategies	About 7 s
Classifier D	Method based on expert experience	About 4 s
Classifier E	Method based on K-means clustering	About 6 s
Classifier F	Method based on surrogate equation in SOM	About 12 s
Classifier G	Method based on measured data in SOM	About 8 s

**Table 9:** (**a**). Detection probabilities of four classifiers under HH polarization. (**b**). Detection probabilities of four classifiers under VV polarization. (**c**). False alarm probabilities of four classifiers under HH polarization. (**d**). False alarm probabilities of four classifiers under VV polarization.

(**a**)					
**Classifier**	**Scenario 1**	**Scenario 2**	**Scenario 3**	**Scenario 4**	**Scenario 5**
ClassifierA	0.85	0.81	0.80	0.76	0.72
ClassifierB	0.85	0.76	0.75	0.72	0.69
ClassifierC	0.86	0.74	0.73	0.70	0.59
ClassifierD	0.83	0.77	0.77	0.72	0.66
ClassifierE	0.80	0.69	0.68	0.66	0.62
ClassifierF	0.85	0.81	0.81	0.76	0.73
ClassifierG	0.75	0.66	0.78	0.63	0.61
(**b**)					
**Classifier**	**Scenario 1**	**Scenario 2**	**Scenario 3**	**Scenario 4**	**Scenario 5**
ClassifierA	0.86	0.82	0.83	0.80	0.75
ClassifierB	0.86	0.79	0.81	0.78	0.72
ClassifierC	0.88	0.75	0.75	0.72	0.61
ClassifierD	0.85	0.79	0.80	0.76	0.69
ClassifierE	0.82	0.70	0.70	0.68	0.66
ClassifierF	0.86	0.82	0.84	0.80	0.76
ClassifierG	0.77	0.67	0.80	0.65	0.63
(**c**)					
**Classifier**	**Scenario 1**	**Scenario 2**	**Scenario 3**	**Scenario 4**	**Scenario 5**
ClassifierA	0.05	0.05	0.12	0.10	0.15
ClassifierB	0.05	0.06	0.16	0.13	0.21
ClassifierC	0.07	0.07	0.22	0.17	0.28
ClassifierD	0.06	0.06	0.18	0.14	0.23
ClassifierE	0.06	0.06	0.21	0.15	0.25
ClassifierF	0.05	0.05	0.11	0.10	0.14
ClassifierG	0.08	0.07	0.20	0.19	0.27
(**d**)					
**Classifier**	**Scenario 1**	**Scenario 2**	**Scenario 3**	**Scenario 4**	**Scenario 5**
ClassifierA	0.06	0.06	0.13	0.11	0.16
ClassifierB	0.07	0.07	0.18	0.15	0.22
ClassifierC	0.08	0.08	0.24	0.18	0.31
ClassifierD	0.07	0.07	0.21	0.15	0.25
ClassifierE	0.07	0.07	0.22	0.16	0.28
ClassifierF	0.06	0.06	0.13	0.11	0.15
ClassifierG	0.08	0.07	0.23	0.21	0.29

## Data Availability

The raw data supporting the conclusions of this article will be made available by the authors on request.
